# The relationship between liver function and neurophysiological factors in depressed individuals: a cross-sectional study using an integrated “East meets West” medicine approach

**DOI:** 10.3389/fpsyt.2023.1159785

**Published:** 2023-05-10

**Authors:** Jiajia Ye, Yunying Yu, Raymond C. K. Chung, Xiaowen Lian, Xin Wang, Wai Ming Cheung, Hector W. H. Tsang

**Affiliations:** ^1^Department of Rehabilitation Sciences, The Hong Kong Polytechnic University, Hung Hom, Hong Kong SAR, China; ^2^Department of Rehabilitation Assessments, Rehabilitation Hospital Affiliated to Fujian University of Traditional Chinese Medicine, Fuzhou, China; ^3^Department of Sleep Medicine, Rehabilitation Hospital Affiliated to Fujian University of Traditional Chinese Medicine, Fuzhou, China; ^4^Department of Clinical Laboratory, Rehabilitation Hospital Affiliated to Fujian University of Traditional Chinese Medicine, Fuzhou, China; ^5^Faculty of Education, The University of Hong Kong, Pokfulam, Hong Kong SAR, China; ^6^Research Centre for Chinese Medicine Inovation, The Hong Kong Polytechnic University, Hung Hom, Hong Kong SAR, China

**Keywords:** traditional Chinese medicine (TCM), depression, liver function, cortisol, heart rate variability (HRV), the hypothalamic–pituitary–adrenal (HPA) axis

## Abstract

**Introduction:**

Depression is a common mental disorder worldwide. The pathology of depression may involve the dysregulation of neurotransmitters and immunity and produce genetic and environmental effects. Traditional Chinese Medicine (TCM) has been practiced for several thousand years and has a different understanding of depression compared to Western medicine. However, this approach has not been widely accepted by scientific communities as TCM mainly focuses on clinical practice.

**Methods:**

In this study, we conducted a cross-sectional study among 100 participants in a rehabilitation hospital to analyze the plausible pathways linking TCM-based liver function and depression, which we hypothesized in a prior theoretical review.

**Results:**

A significant relationship between adrenocorticotropic hormone and TCM-based liver function was found (*r* = 0.211, *p* = 0.041). Cortisol was significantly associated with norepinephrine (*r* = 0.243, *p* = 0.015) and adrenocorticotropic hormone (*r* = 0.302, *p* < 0.001). A positive significant relationship was also found between norepinephrine and adrenocorticotropic hormone (*r* = 0.272, *p* < 0.001). There was no significant relationship between the ratio from low frequency to high frequency and TCM-based liver function (*p* = 0.690).

**Discussion:**

These results suggest that TCM-based liver function can be interpreted using the hypothalamic-pituitary-adrenal axis. This is a pioneering study to examine the mechanisms of depression in relation to liver function by integrating Eastern and Western medical approaches. The findings of this study are valuable for a deeper understanding of depression and public education.

## 1. Introduction

Depression is a common psychiatric disorder characterized by a loss of interest and energy as well as a depressed mood, which causes much disability and mortality worldwide (1). Compared to healthy individuals, depressed individuals may experience the worse quality of life, sleep disorders, fluctuations in body weight, and a reduced life expectancy, which creates a heavy economic burden. Following the increasing rates of depression, developing a better understanding of this illness together with efforts to prevent it and reduce the growing economic burden that results from it, have been recognized as public health priorities.

Traditional Chinese medicine (TCM) has been applied in clinical practice for more than 2000 years in China (2). In contrast to Western medicine, TCM adopts a holistic approach as its theoretical foundation, which has evolved based on accumulated clinical experiences (3). Worldwide, more than 200 million patients have received TCM therapies, including acupuncture, massage, and Chinese herbs (4). Previous systematic reviews suggested that TCM therapies significantly improved depressive syndromes (5–9). Although the efficacy of TCM is obvious, its mechanisms of TCM for treating diseases are still unknown to scientific communities as TCM is mainly based on clinical practice instead of solid scientific evidence; therefore, more research is urgently needed to evaluate the mechanisms of TCM for treating diseases according to modern scientific approaches.

**Figure 1:**
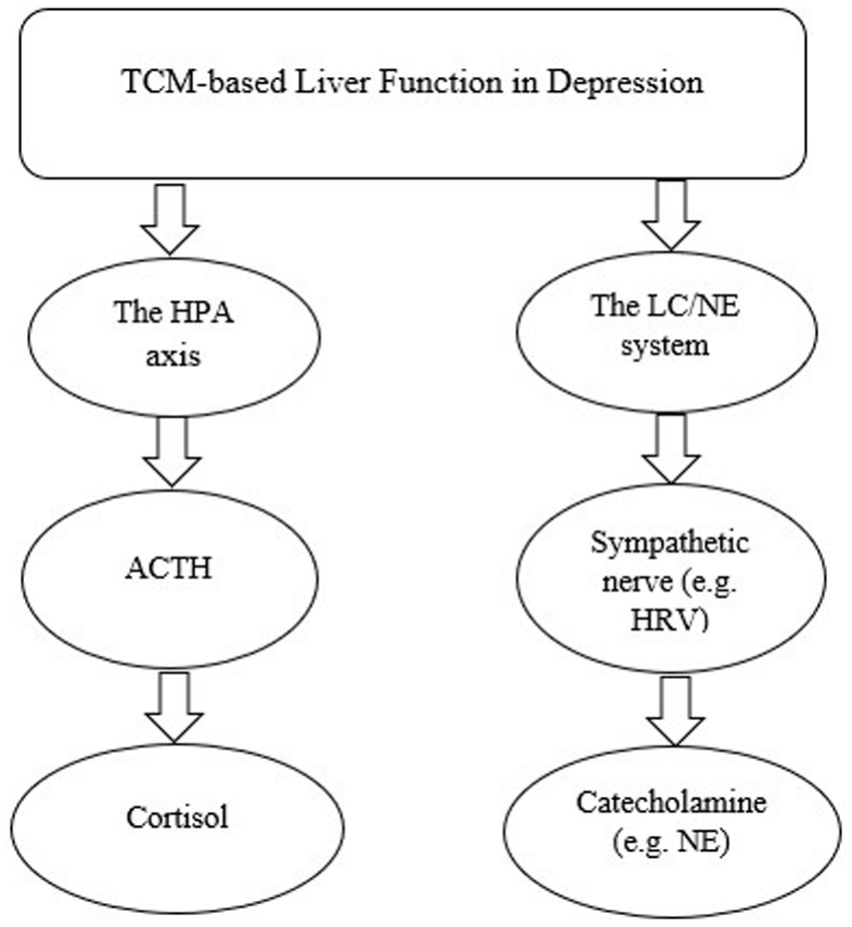
The hypothesized models to interpret TCM-based liver function in depression. TCM, Traditional Chinese medicine; HPA, hypothalamic–pituitary–adrenal; LC/NE, locus coeruleus-norepinephrine.

Two plausible pathological pathways linking TCM-based liver function and depression have been proposed in our prior theoretical review (4) (Figure 1). Based on TCM theory, liver function is first affected when there is an emotional change. By contrast, Western medicine suggests that depression has a profound relationship with neurotransmitters, for example, norepinephrine, epinephrine, and serotonin (10). The previous theoretical review suggested that there are plausible mechanisms of depression in TCM according to the Western medical approach (4). However, there is no experimental study to support this theory. Therefore, the aim of this study is to test and validate the plausible pathways linking TCM-based liver function to depression based on the integration of Eastern and Western medicine, while also obtaining a deeper understanding of depression.

## 2. Materials and methods

### 2.1. Participants and sampling size

Potential participants were invited to join this research study by a member of the research team from the Rehabilitation Hospital in Fujian, China between October 2020 and October 2021. A qualified research assistant who received diagnostic training from a certified clinical psychiatrist and a TCM practitioner explained the research and conducted an initial screening for those who showed an interest in participating. The criteria for inclusion in the study involved the following: (1) were aged between 18 and 65; (2) had been diagnosed as suffering from a current episode of depression by a psychiatrist based on the DSM-5 criteria; (3) had been diagnosed as experiencing symptoms of liver Qi stagnation syndrome by the International Medicine of Traditional Chinese Medicine (11); (4) had a baseline score of 12 or higher on the 17-item Hamilton Rating Scale for Depression (HRSD_17_) (12); (5) scored 20 or higher on the Montreal Cognitive Assessment (MoCA); and (6) were willing to give consent by signing a written informed consent form. Participants were excluded if they fit any of the following criteria: (1) were primarily diagnosed with an illness other than depression; (2) were pregnant or lactating women; (3) were experiencing substance abuse or drug dependence; and (4) were enacting acute suicidal or violent behavior. All eligible participants were asked to sign their informed consent forms, which were formulated according to the declaration of Helsinki. This study was approved by the local Research Ethics Committee (approval number: 2020YJS-003-01) and the trial was registered in the Chinese Clinical Trial Registry (ChiCTR1900027222).

The calculation of the sample size was based on the rule of thumb that for each parameter, 20 subjects are recommended (13). Therefore, a total number of 100 participants were needed for this study.

### 2.2. Procedures for data collection

Participants’ demographic and anthropometric data—including their age, gender, education, height, body weight, and marital status—were recorded after the informed consent form had been signed. After recording their personal information, eligible participants were asked to abstain from coffee, tea, and alcohol for 24 h before 9 a.m. of the day of measurement. Blood samples were obtained each day for 1 week after the initial screening by a certified phlebotomist and heart rate variability (HRV) was measured at the same time interval on the same day as the blood samples were taken. All testing was conducted in a quiet laboratory setting to minimize distraction.

### 2.3. Outcome measurements

#### 2.3.1. Liver function in TCM

The severity of liver Qi stagnation was assessed according to the “guiding principles of clinical research on new drugs of Traditional Chinese Medicine” (14, 15). The syndromes were described as follows: (1) major syndromes, including mental depression, frustration, pessimism, feeling world-weary, and sighing often; (2) secondary syndromes, including poor memory, insomnia, irritability, belching, hiccups, feeling restless, pain, abdominal distension, and a foreign body sensation in the throat; (3) a coating on the tongue, for example, a pink tongue with a thin white coating; and (4) a pulse sign, for example, a wiry pulse. The items in major syndromes were rated on a seven-point scale ranging from 0 to 6 and the items in secondary syndromes were rated on a four-point scale ranging from 0 to 3. The higher the total score, the more severe the liver Qi stagnation.

#### 2.3.2. Neurophysiological biomarkers

NE, adrenocorticotropic hormone (ACTH), and cortisol levels were collected between 9 and 11 a.m. from participants. A 5-mL tube with EDTA-Na_2_ was used to collect the whole blood from a vein in the cubital fossa, which was then centrifuged for 15 min at 1,000×*g* at 4°C. After 30 min, the isolated plasma was collected, and the plasma sample was then stored at −20°C until analysis. When the plasma was ready to process, the frozen plasma was thawed, and Enzyme-linked immunosorbent assay (ELISA) kits (Elabscience Biotechnology Co. Ltd., Wuhan, China) were used to determine the concentrations of NE, ACTH, and cortisol. The NE assay had a sensitivity of 0.19 ng/mL with an intra-assay and inter-assay variance of less than 10%. The ACTH assay had a sensitivity of 9.38 pg/mL with an intra-assay and inter-assay variance of less than 7%. The cortisol assay had a sensitivity of 2.92 ng/mL with an intra-assay and inter-assay variance of less than 9%. All measurements were taken in line with the instructions from the manufacturer.

#### 2.3.3. Heart rate variability

Participants were instructed to sit in a chair for a rest period of 15 min at a room temperature of 26°C before measurement and were told to relax and breathe normally during measurement. HRV data was acquired using a battery-operated portable HRV device (Check MyHeart™, Daily Care Biomedical, Taiwan) (16).

Data was obtained from a 5-min ECG with a sampling rate of 250/s. Two electrodes were placed on both sides of the inner forearms. The raw ECG data was exported to a PC using a USB cable for subsequent analysis (17). Frequency-domain analysis was performed using the non-detrend method of fast Fourier transformation (FFT). A ratio from LF (low frequency) to HF (high frequency) was selected since it represents sympathovagal balance or reflects sympathetic modulations (18). Participants received 100 HKD in compensation for travel expenses after completing all of the assessments.

### 2.4. Data analysis

Descriptive analyses were used to measure the demographic data and frequency analyses were employed to measure the enumeration data. Continuous variables were described using the mean and standard deviation (SD). The Shapiro–Wilk test was used to test data normality for each outcome variable. Log10 transformation was performed to meet the assumptions of normality if the variables did not follow the normal distribution. The Pearson correlation coefficient of cortisol, ACTH, NE, HRV, and TCM-based liver function were investigated. Statistical analyses were performed using the SPSS version 25.0 (SPSS Inc., United States).

**Figure 2:**
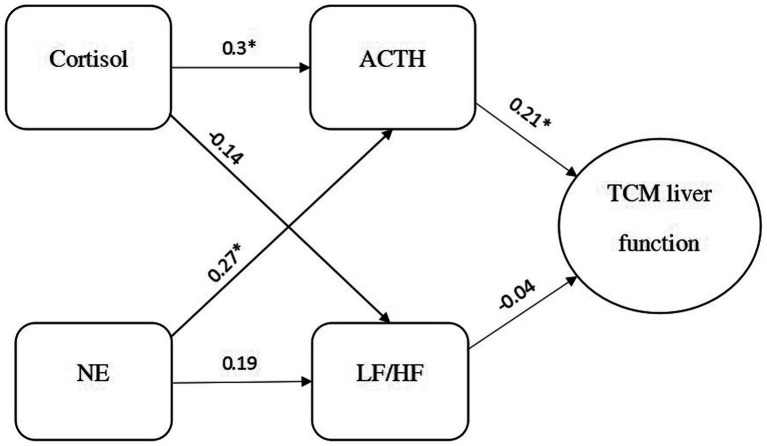
Path analysis model with standardized regression coefficients among 100 participants. The goodness-of-fit of the hypothetical path model: *χ*^2^ = 4.689, df = 3, *p =* 0.196, *χ*^2^/df = 1.563, CFI = 0.947, NFI = 0.888, and RMSEA = 0.075. ACTH, adrenocorticotropic hormone; TCM, Traditional Chinese medicine; NE, norepinephrine; *χ*^2^, chi-square; LF/HF, a ratio from low frequency to high frequency; df, degree of freedom; CFI, comparative fit index; NFI, normed fit index; RMSEA, root mean square error of approximation.

Figure 2 depicts the hypothesized model that explores both the direct and indirect effects of neurophysiological biomarkers on the severity of TCM-based liver dysfunction in depressed patients using path analysis. The maximum-likelihood estimation was used to test the fit of the hypothesized model. The variables and the direction of the relationship were based on an earlier study (4). Chi-square (*χ*^2^), the root mean square error of approximation (RMSEA), the normed fit index (NFI), and the comparative fit index (CFI) were selected to evaluate the goodness of fit of the model. A value of 0.08 or below for RMSEA was considered an “adequate fit” (19) and a value of 0.9 or above for both CFI and NFI was regarded as a “good fit.” A non-significant likelihood ratio of *χ*^2^ and the degree of freedom (df) test suggests a good model fit, but as it is sensitive to sample size, then an *χ*^2^/df ratio of 3 or less indicates an acceptable fit (20, 21). The path model was applied using the statistical software IBM SPSS AMOS version 25.0. The level of statistical significance was set at *p* < 0.05.

## 3. Results

### 3.1. Demographic characteristics of the participants

As shown in Table 1, a total of l00 subjects participated in this study. The mean age, height, and weight of the participants were 45.06 ± 14.37, 164.20 ± 8.96, and 59.13 ± 9.79, respectively. The majority of the participants were female (70%) and 81% of the participants were married. Approximately 70% of participants were highly educated, having received an education for more than 12 years (22).

**Table 1:** Demographic data of participants.

Characteristics	Mean	SD
Age, year	45.06	14.37
*Gender, n (%)*		
Male	30 (30%)	
Female	70 (70%)	
Height (cm)	164.20	8.96
Weight (kg)	59.13	9.79
*Education status, n (%)*		
Low (< 9 years)	10 (10%)	
Medium (9–12 years)	20 (20%)	
High (>12 years)	70 (70%)	
*Marital status, n (%)*		
Single	19 (19%)	
Married	81 (81%)	

### 3.2. Normality of research variables

All of the variables of ACTH, NE, cortisol, and HRV in this study did not meet the assumption of normal distribution. Thus, the log10 transformation was performed for subsequent analysis. The mean and SD for all variables are outlined in Table 2.

**Table 2:** Correlations between neurophysiological biomarkers and TCM liver function.

	Cortisol	LF/HF	NE	ACTH	TCM liver function
Cortisol	1.000	−0.140	0.243^*^	0.302^*^	−0.002
LF/HF	–	1.000	0.187	0.163	−0.039
NE	–	–	1.000	0.272^*^	0.136
ACTH	–	–	–	1.000	0.211^*^
TCM liver function	–	–	–	–	1.000
Mean	2.489	0.124	0.935	3.116	1.397
SD	0.113	0.427	0.125	0.819	0.080

LF/HF, low frequency/high frequency; NE, Norepinephrine; ACTH, adrenocorticotropic hormone; TCM, traditional Chinese medicine. *p < 0.05.

#### 3.2.1. Relationship between neurophysiological biomarkers and TCM-based liver function

The relationship between ACTH and TCM-based liver function was significant (*r* = 0.211, *p* = 0.041). Cortisol was significantly associated with NE (*r* = 0.243, *p* = 0.015) and ACTH (*r* = 0.302, *p* < 0.001). A positive significant relationship was also found between NE and ACTH (*r* = 0.272, *p* < 0.001). The correlational relationships between all variables are shown in Table 2.

### 3.3. Fitness of the hypothetical path model

Figure 2 presents the results of the path analysis. The goodness-of-fit of the hypothetical path model were *χ*^2^ = 4.689, df = 3, *p =* 0.196, *χ*^2^/df = 1.563, CFI = 0.947, NFI = 0.888, and RMSEA = 0.075. Although one of the indices (NFI = 0.888) was rather unsatisfactory and did not fulfill the basic requirement (NFI < 0.90) in the present study, this model could still be regarded as “acceptable” because the other indices had already fulfilled the requirements following the implementation of recommendations suggested by the Modification Indices (23).

### 3.4. Testing of the plausible neurophysiological pathways and verification of the variable effects

All of the standardized beta coefficients and the total effect of the variables on TCM-based liver function are depicted in Figure 2.

The pathway related to the HPA axis (from cortisol, ACTH to TCM-based liver function) was supported; cortisol had a significant positive correlation with ACTH (*β* = 0.302, *t* = 3.266, *p* = 0.001) and ACTH also had a significant positive relationship with TCM-based liver function (*β* = 0.211, *t* = 2.147, *p* = 0.032) (Figure 2). These findings indicate that cortisol partially affects TCM-based liver function through the mediating effect of ACTH. However, the pathway regarding the LC-NE system (from NE, HRV parameter to TCM-based liver function) was not supported; NE had a marginally significant association with the LF/HF ratio (*β* = 0.187, *t* = 1.840, *p* = 0.066) and there was no significant relationship between the LF/HF ratio and TCM-based liver function (*β* = −0.039, *t* = −0.398, *p* = 0.690) (Figure 2).

In addition, a positive significant correlation between NE and ACTH was found (*β* = 0.272, *t* = 2.946, *p* = 0.003). An insignificant relationship was observed between cortisol and the LF/HF ratio (*β* = 0.-140, *t* = −1.385, *p* = 0.166).

## 4. Discussion

The present study tested two neurophysiological pathways based on the integration of Eastern and Western medicine using path analysis. These two neurophysiological pathways were: (1) the HPA axis (from cortisol, ACTH to TCM-based liver function); (2) the locus coeruleus-norepinephrine (LC/NE) system (from NE, HRV parameter to TCM-based liver function). To our knowledge, this is an innovative study that examines the mechanisms of TCM-based liver function in terms of depression based on an integrated approach of East meets West. The findings of this study corroborated that TCM-based liver function may be interpreted using the HPA axis but not the LC-NE system.

The results of this study showed that cortisol and ACTH were positively correlated with TCM-based liver function in individuals with depression. This finding supported the proposed pathways laid out in our prior theoretical review (4). Previous experimental studies (24–26) suggested that increased cortisol and ACTH had a close link with more severe depressive syndromes. TCM practitioners widely believe that the Chinese herb of Chaihu may target liver function and have an influence on mental illness (27, 28). A study by Zhong Xiaoming et al. (29) found that the Shuyu capsule—extracted from Chaihu—significantly improved the levels of cortisol and ACTH in patients with depression, as demonstrated by the HPA axis. Another study by Xu Teng et al. (30) found that Chaihu significantly reduced the incidence of depressive-like behaviors in rats through regulating the HPA axis. Moreover, a study by Wei Liping et al. (31) highlighted that the Chaihu formula statistically improved the HPA axis, which reduced depression in rats. It is increasingly evident that liver function in TCM has had a close link with the HPA axis in depression. Consistent with the above findings, our results verify that the links between TCM-based liver function and depression can be interpreted using the HPA axis.

Admittedly, NE was marginally correlated with HRV and the relationship between HRV and TCM-based liver function was not found in the present study. A cross-sectional study by Baumert and his colleagues (32) suggested that there was a somewhat positive relationship found between NE and HRV parameters in patients with depression. A randomized controlled study by Davidson et al. (33) found that antidepressants blocked norepinephrine uptake and also lowered HRV parameters. Moreover, another study by Ahrens et al. (34) highlighted that the central noradrenergic function is closely connected to HRV parameters in patients with major depression. The above results supported our current findings that a marginally positive relationship was found between HRV and NE. In contrast, our findings on HRV and TCM-based liver function were not supported by previous experimental studies. A study by Shi et al. (35) suggested that acupuncture treatments targeting acupoints in the liver, heart, and brain—according to TCM—might significantly improve HRV parameters in patients with depression. Another study by Hu Yiting et al. (36) emphasized that the capsules of Shugan Jieyu that target TCM-based liver Qi stagnation significantly improved the standard deviation of NN intervals (SDNN) as well as the low frequency (LF) and the high frequency (HF) of the HRV parameters in individuals with depression. Furthermore, a study by Yi Lin et al. (37) indicated that the decoction of Shugan increased HF in those depressed patients who had a differentiation in the syndrome of liver Qi stagnation. Significantly, LC innervating and receiving information from the spinal cord and different brain areas is at the center of the synthesizing adrenergic nerve in the brain (38). There is evidence that LC can increase the synthesis of NE and enhance the activity of the sympathetic nerves through activating the HPA axis in stress-related disorders (10), for example, for depression and anxiety. Based on TCM, liver dysfunction and depression may have similar clinical syndromes as the increased activity of the sympathetic nerves, such as irritability, increased muscle tension and heart rate, and poor digestion. Moreover, TCM treatments targeting the regulation of the liver can significantly improve depressive syndromes. Nevertheless, our results did not fully support this view—this may have been caused by the insufficient sample size in this study. Therefore, more studies with a larger sample size will be necessary to examine the relationship between sympathetic nerves and TCM-based liver function.

Interestingly, our results showed a positive correlation between NE and ACTH but a non-significant negative correlation between cortisol and the LF/HF ratio. ACTH plays a vital role in stimulating the adrenal glands to release androgens and to increase the secretion of catecholamine, including NE and adrenaline (E) (39, 40). Thus, the relationship between NE and ACTH may provide further insight to further interpret TCM-based liver function. Thus, further studies are needed to analyze this potential pathway.

## 5. Conclusion

Depression is a widespread mental disorder that causes a heavy economic burden on our societies. TCM and Western medicine have different views on depression. This is the first study to examine the pathology of depression regarding TCM-based liver function through Western scientific approaches. TCM-based liver function may be interpreted by the HPA axis, and the LC-NE system may partly explicate TCM-based liver function.

## 6. Limitations

The present study has some limitations that readers need to be aware of. First, the participants were recruited from only one hospital. Thus, the results might not be generalizable to the entire population who suffer from depression with TCM-based liver dysfunction. Second, the majority of participants were female (70%), which may produce a gender bias. Third, the present study involved only a cross-sectional data analysis—that is, the causal relationship between TCM-based liver function and the HPA axis could not be established based on the data from this study. Future studies using a longitudinal design are highly recommended to analyze the casual relationship of variables involved in the pathways of depression based on TCM theory. Fourth, the sample size may not be large enough in this study. Some researchers recommended that the minimum sample size for path analysis using AMOS software was 200 (41). Therefore, further experimental studies with a larger sample size that will analyze the pathological models based on the integration of Eastern and Western medicine are highly recommended. Fifth, this study only tested the hypothesized models regarding TCM-based liver function, further studies are strongly required to test other plausible models linking TCM-based spleen and heart functions. Lastly, only participants experienced both current depression and TCM-based liver dysfunction were recruited in this study, thus, the variation of TCM-based liver function among these subjects is small. That is difficult to observe significant results due to the small variation. Further research may include both depression and non-depression participants to investigate the relationship between potential biomarkers and TCM-based organ function.

## Data availability statement

The raw data supporting the conclusions of this article will be made available by the authors, without undue reservation.

## Ethics statement

The studies involving human participants were reviewed and approved by Rehabilitation hospital affiliated to Fujian University of Traditional Chinese Medicine. 2020YJS-003-01. The patients/participants provided their written informed consent to participate in this study.

## Author contributions

The project was conceived and designed by WC and HT. JY performed the search and drafted the initial manuscript. JY, YY, XL, RC, and XW contributed to data analysis and interpretation. All authors contributed to the article and approved the submitted version.

## Funding

This work was funded by the Bright Future Charitable Foundation of Hong Kong, China (grant no. P0030920).

## Conflict of interest

The authors declare that the research was conducted in the absence of any commercial or financial relationships that could be construed as a potential conflict of interest.

## Publisher’s note

All claims expressed in this article are solely those of the authors and do not necessarily represent those of their affiliated organizations, or those of the publisher, the editors and the reviewers. Any product that may be evaluated in this article, or claim that may be made by its manufacturer, is not guaranteed or endorsed by the publisher.
